# Functional Dystonia: Differentiation From Primary Dystonia and Multidisciplinary Treatments

**DOI:** 10.3389/fneur.2020.605262

**Published:** 2021-02-04

**Authors:** Lucy Frucht, David L. Perez, Janet Callahan, Julie MacLean, Phillip C. Song, Nutan Sharma, Christopher D. Stephen

**Affiliations:** ^1^Faculty of Arts and Sciences, Harvard University, Boston, MA, United States; ^2^Cognitive Behavioral Neurology Unit, Department of Neurology, Massachusetts General Hospital and Harvard Medical School, Boston, MA, United States; ^3^Functional Neurological Disorder Research Program, Department of Neurology, Massachusetts General Hospital and Harvard Medical School, Boston, MA, United States; ^4^Neuropsychiatry Division, Department of Psychiatry, Massachusetts General Hospital and Harvard Medical School, Boston, MA, United States; ^5^MGH Institute of Healthcare Professionals, Massachusetts General Hospital and Harvard Medical School, Boston, MA, United States; ^6^Occupational Therapy Department, Massachusetts General Hospital and Harvard Medical School, Boston, MA, United States; ^7^Department of Otolaryngology, Massachusetts Eye and Ear Infirmary, Harvard Medical School, Boston, MA, United States; ^8^Dystonia Center and Movement Disorders Unit, Department of Neurology, Massachusetts General Hospital and Harvard Medical School, Boston, MA, United States

**Keywords:** dystonia, functional dystonia, functional movement disorder, functional neurological disorder, therapeutics

## Abstract

Dystonia is a common movement disorder, involving sustained muscle contractions, often resulting in twisting and repetitive movements and abnormal postures. Dystonia may be primary, as the sole feature (isolated) or in combination with other movement disorders (combined dystonia), or as one feature of another neurological process (secondary dystonia). The current hypothesis is that dystonia is a disorder of distributed brain networks, including the basal ganglia, cerebellum, thalamus and the cortex resulting in abnormal neural motor programs. In comparison, functional dystonia (FD) may resemble other forms of dystonia (OD) but has a different pathophysiology, as a subtype of functional movement disorders (FMD). FD is the second most common FMD and amongst the most diagnostically challenging FMD subtypes. Therefore, distinguishing between FD and OD is important, as the management of these disorders is distinct. There are also different pathophysiological underpinnings in FD, with for example evidence of involvement of the right temporoparietal junction in functional movement disorders that is believed to serve as a general comparator of internal predictions/motor intentions with actual motor events resulting in disturbances in self-agency. In this article, we present a comprehensive review across the spectrum of FD, including oromandibular and vocal forms and discuss the history, clinical clues, evidence for adjunctive “laboratory-based” testing, pathophysiological research and prognosis data. We also provide the approach used at the Massachusetts General Hospital Dystonia Center toward the diagnosis, management and treatment of FD. A multidisciplinary approach, including neurology, psychiatry, physical, occupational therapy and speech therapy, and cognitive behavioral psychotherapy approaches are frequently required; pharmacological approaches, including possible targeted use of botulinum toxin injections and inpatient programs are considerations in some patients. Early diagnosis and treatment may help prevent unnecessary investigations and procedures, while facilitating the appropriate management of these highly complex patients, which may help to mitigate frequently poor clinical outcomes.

## Introduction

Dystonia is a common movement disorder involving sustained muscle contractions, often resulting in twisting and repetitive movements and abnormal postures related to an imbalance of agonist and antagonist muscles (1). Dystonia may indicate the presence of many neurological disorders—involving a primary dystonia, which may be the sole feature (isolated) or in combination with other movement disorders (combined dystonia), or as a feature of another neurological process (secondary dystonia) (1). Furthermore, although many forms of dystonia do not have any identified genetic underpinnings (idiopathic dystonia), several genetic forms have been identified, most commonly DYT-TOR1A (DYT1), DYT-THAP1 (DYT6), and DYT-GCH1 (dopa-responsive dystonia) (2). Dystonia is common, with an estimated prevalence of up to 1 in 1,000 individuals, which is notable, given diagnostic challenges frequently leading to misdiagnosis or under-diagnosis (3, 4). The anatomical basis and pathophysiology of dystonia is unknown, with neuropathology generally not revealing brain degeneration except in the case of X-linked dystonia parkinsonism (DYT-TAF1 [DYT3]) (2). The current hypothesis is that dystonia is a disorder of brain networks, including the basal ganglia, cerebellum, thalamus and cortex, resulting in abnormal neural motor programs (5). Table 1 illustrates the range of clinical presentations in common genetic primary dystonia, including the phenotype, mode of inheritance and typical age of onset.

**Table 1:** Common genetic dystonia subtypes [adapted from Klein et al. (6) and Balint et al. (2)].

**Classification**		**Designation/Gene locus**	**Onset**	**Pattern of inheritance**	**Dystonia distribution**	**Other relevant features**
Isolated dystonia		*DYT-TOR1A (DYT1)*	C	AD	Generalized	• **Most common genetic dystonia** • More common in Ashkenazi Jewish ancestry • Focal onset, frequently in the lower limbs and generalizes • Reduced penetrance • **DBS highly effective** **(****7**, **8****)**
		*DYT-THAP1 (DYT6)*	A/C	AD (rarely AR)	Neck, limbs, orofacial, and larynx	• Often prominent cranial involvement • **DBS beneficial** **(****7**, **8****)**
		*DYT-ANO3 (DYT24)*	A/C	AD	Neck, larynx, orofacial, and upper limbs	• Onset typically cervical
		*DYT-GNAL (DYT25)*	A	AD (rarely AR)	Neck limbs, orofacial, and larynx	
Combined dystonia	Parkinsonism	*DYT/PARK-TAF1 (DYT3)*	A	XR	Orofacial, neck, limbs, and trunk	• Filipino ancestry, often to Panay Island • Wide phenotypic spectrum ranging from severe generalized dystonia, pure parkinsonism or combination • Unique dystonic parkinsonian gait (9) • MRI with striatal atrophy • **DBS beneficial** **(****7**, **8****)**
		*DYT-GCHI (DYT5a)*	C	AD (rarely AR)	Limbs and trunk	• **Dopa-responsive** • Diurnal variation (worse in evenings) • Spasticity • Familial “cerebral palsy”
		*DYT-TH (DYT5b)*	C	AR	Limbs, trunk, and orofacial	• **Dopa-responsive** • Diurnal variation • Gait disorder • Myoclonus • Spasticity • May be associated with oculogyric crises • **DBS beneficial** **(****7**, **8****)**
		*DYT-SPR*	C	AR (rarely AD)		• **Dopa-responsive** • Diurnal variation • Intellectual/developmental delay • High CSF biopterin/dihydrobiopterin • May be associated with oculogyric crises
		*DYT-ATP1A3 (DYT12)*	A/C	AD	Orofacial, cervical, larynx, and limbs	• Clinical heterogeneity: - Alternating hemiplegia of childhood - Rapid onset dystonia parkinsonism · Common bulbar involvement - Cerebellar ataxia, areflexia, pes cavus, optic atrophy (CAPOS) • Sudden onset after infection/febrile illness • Fluctuating course • Exacerbations with fever, physical stress, alcohol • Chorea • May have seizures
		*DYT-PRKRA (DYT16)*	C	AD	Orofacial, larynx, neck, trunk, and limbs	• Hyperreflexia
	Myoclonus	*DYT-SGCE (DYT11)*	C	AD	Neck, upper limbs, and orofacial	• Alcohol dependence (improves myoclonus) • Neuropsychiatric symptoms • **DBS beneficial** **(****7**, **8****)**
		*DYT- KCTD17 (DYT26)*	A/C	AD	Cranial and cervical	
		*DYT- KMT2B (DYT28)*	C	AD	Orofacial, larynx, neck, limbs, and trunk (may have myoclonus)	• Microcephaly • Short stature • Neuropsychiatric symptoms • Intellectual/developmental delay • Oculomotor apraxia • “Cerebral palsy” • **DBS beneficial** **(****7**, **8****)**
	Paroxysmal	*PxMD-PRRT2 (DYT10/19)*	C	AD		• Paroxysmal kinesogenic dyskinesia (PKD) • Attacks triggered by sudden voluntary movements, stress, startle, sleep deprivation • Migraine (may be hemiplegic) • May have epilepsy
		*PxMD-PNKD (DYT8/20)*	C	AD		• Paroxysmal non-kinesogenic dyskinesia (PNKD) with choreoathetosis, ballismus • Attacks triggered by alcohol, caffeine stress, hunger, fatigue, tobacco
		*PxMD-SLC2A1 (DYT9/18)*	C	AD	Legs most commonly	• Paroxysmal exertional dyskinesia (PED) with choreoathetosis
		*PxMD-ECHS1*	C	AR		• Paroxysmal exertional dyskinesia (PED) • Severe developmental delay • Infantile encephalopathy with choreoathetosis • Optic atrophy • Cardiomyopathy • Sensorineural hearing loss
		ADFLE (CHRNA4)	C	AD		• Paroxysmal hypnogenic dyskinesias
	Other	*DYT-TUBB4A (DYT4)*	A/C	AD	Orofacial, larynx, neck, and limbs	• “Whispering” dysphonia • “Hobby horse” gait • Ptosis, edentulous, facial atrophy
		*DYT- MECR (DYT29)*	C	AR	Generalized	• Optic Atrophy • Basal ganglia abnormalities
		*CHOR/DYT-ADCY5*	C	AD, de novo, rare AD	Generalized	• Axial hypotonia • Developmental delay • Facial twitching • Chorea • Myoclonus • Oculomotor apraxia • Triggered by sleep transitions, emotional stress, illness, sneezing, caffeine
		*ACTB*	C	AD	Generalized	• Sensorineural deafness • Intellectual/developmental delay • Dysmorphic facies • **DBS beneficial** **(****7**, **8****)**

*AD, autosomal dominant; AR, autosomal recessive; XR, X-linked recessive; A, adult onset; C, childhood onset; CSF, cerebrospinal fluid; DBS, deep brain stimulation; ADFLE, autosomal dominant frontal lobe epilepsy. Bolding in table is used for emphasis of particularly important features*.

Functional dystonia (FD) may resemble other forms of dystonia (OD) but has a different pathophysiology (10, 11), as a subtype of functional movement disorders (FMD) under the more general classification of functional neurological disorders (FND) (12). The study of FND is rapidly developing, as these are common but still incompletely understood disorders at the interface between neurology and psychiatry (13). FD is both phenotypically and phenomenologically diverse, with frequent mixed presentations with other FMDs and/or other functional symptoms and can present from childhood to late adulthood (14). Thus, although FD is the second most common FMD, it is among the most diagnostically challenging FMD subtypes (11). Distinguishing between FD and OD is important, as the management of these disorders is different and appropriate treatment may lead to symptom resolution in FD (14, 15). We present a comprehensive (long-form) review of the historical context, clinical clues and features differentiating FD from OD across the spectrum of FD subtypes (including pediatric FD), evidence for adjunctive “laboratory-based” testing, and pathophysiology. In doing so, we outline the approach used at the Massachusetts General Hospital (MGH) Dystonia Center toward the diagnosis, management and treatment of this complex set of disorders.

## Historical Context

Even after over a century of examination and consideration of its presentation, symptoms, and diagnostic criteria, FD remains a challenging movement disorder for neurologists to diagnose, and patients to understand (16). For this reason, the legitimacy of FD has historically been heavily debated. For the better part of the twentieth century, FD was considered purely a manifestation of “conversion disorder/hysteria,” with physical symptoms and signs believed by physicians to be entirely psychiatric in nature (17). However, over time, given growing evidence of genetic inheritance and its pathophysiological underpinnings, dystonia was framed as an “organic” neurological disorder (18). Given this realization, the pendulum then swung in the opposite direction, whereby FD was felt to be rarely present (19). Time has eventually confirmed the presence of FD, taking its place alongside traditional dystonia but inherent difficulties remain, including that there can be an overlap in these distinct disorders, adding to the diagnostic and clinical challenges (20). Hence to understand FD in the present, we first must look to the past.

One of the founders of neurology, Jean-Martin Charcot's description of “hysteria” mirrored the typical presentation of dystonia that doctors faced in the clinic decades later (21). Early case reports of what is now known to be early-onset generalized torsion dystonia involved diagnostic impressions punctuated by a history of psychiatric issues and “hysterical” symptoms (21). However, in 1911, Hermann Oppenheim provided the first documentation of dystonia and coined the term. After seeing several similar unusual cases, he published his observations and theories about their commonalities, assigning them the label, “dystonia musculorum deformans” (22). He struggled over the disorder's pathogenesis, and ultimately concluded, “I therefore believe that the affliction we are dealing with today is not a neurosis but rather originates from subtle changes in the central nervous system, in special areas that control or influence the muscle tone and that it will be possible sooner or later to discover the pathological-anatomical cause” (22). He believed this first description of generalized dystonia to be a neurological condition, which appeared familial in some cases but could not distinguish a pathological cause (22). There was similar dispute in forms of focal dystonia. In cervical dystonia (torticollis), the turning of the head was debated to be a peripheral nerve disorder, while the common presence of a “geste antagoniste,” or sensory trick (a now typical and widely acknowledged finding suggestive of dystonia), was given as evidence by Charcot of its psychological origins (21). Writer's cramp, a focal hand dystonia, received a similar fate, where the eminent neurologist, Guillaume Duchenne described this as “spasme fonctionnel” (23), a neurological disorder but others focused on the etiological role of personality traits (21, 23). Contemporaneous with Oppenheim, neurologist Sigmund Freud was gaining traction for his theories and practices of psychoanalysis to treat “conversion disorder” (24). The symptoms of hysteria resembled neuroses but had unknown pathology and were felt to be usually triggered by an emotional or traumatic event, with physical manifestations resulting from internal psychological conflict (25). Therefore, dystonia came to be regarded as a form of “conversion disorder”; a psychological phenomenon, which was treated with psychotherapy (21).

Through the mid-twentieth century, dystonia presentations such as blepharospasm were treated primarily with long-term psychotherapy and other, sometimes experimental psychiatric treatments (26). Although met with limited success, attempts at more medicalized treatments, such as eye drops and carbon dioxide treatments, proved futile (27). In one case, a man with blepharospasm who claimed no psychiatric history improved immediately with the administration of a central nervous system stimulant, suggesting a relationship between his dystonia and underlying depression (27). In addition, cervical dystonia was treated with psychotherapy, anxiolytics, behavioral therapy or aversive therapies (28). However, these conventions were challenged by a growing body of evidence culminating with studies in the 1960s identifying the neuropathologic basis of dystonia (29). Firstly, psychotherapy did not produce the expected benefits seen in other traditional forms of “hysteria” (30). In addition, surgery to the thalamus or globus pallidus [typical targets for current deep brain stimulation (DBS) in OD] yielded substantial clinical benefit, and the first animal model of dystonia, following lesioning of the basal ganglia, was produced in 1965 by Denny-Brown, one of the pioneers of American neurology (10, 31, 32).

By the 1970s, David Marsden popularized dystonia's neurological features and sidelined FD as a markedly uncommon entity (21). He described several features leading to dystonia being erroneously considered as “psychogenic,” including the at times bizarre phenomenology of the movements, the inexplicable task-specificity, the presence of sensory tricks, typical worsening with stress and anxiety, and lack of abnormalities on diagnostic testing (33). As such, many doctors viewed a FD diagnosis as exceedingly rare, or perhaps did not even exist (19). A study from 1978 examined the differences in patients with idiopathic dystonia, having the potential to be characterized as “psychogenic,” and those whose dystonia had a known cause or family history and ultimately determined that only 1 out of 85 cases were “psychogenic” in origin (34). With the understanding of the frequency of psychiatric symptoms in dystonia, this led to a shift in favor of a neurological definition of dystonia (35). Subsequently, in the 1970s and 1980s, there were a deluge of cases detailing misdiagnosis of dystonia as “psychogenic” and highlighting the medical and legal perils of this formulation (34, 36, 37).

Later, neurological cases of “psychogenic” dystonia responding to psychotherapeutic treatment were reported in the late 1970's and early 1980's (21). This again came from leaders in the field, and while Stanley Fahn reported the first cases of FD, he still considered this a rare entity (34, 38). Following further evidence from an expanding number of cases, Fahn and Williams set out the first diagnostic definition of FD (39).

Despite the clarification brought by initial and subsequently updated diagnostic criteria (40), diagnosis of FD remains challenging to general neurologists and movement disorders specialist alike, with poor interrater reliability (41, 42). Additionally, the shift from “psychogenic” to “functional” dystonia occurring in the twenty-first century (43) seeks to provide an etiologically neutral framing of this condition—one that acknowledges that this neuropsychiatric condition sits at the intersection of neurology and psychiatry (44). Current research efforts therefore aim to identify features which reliably differentiate FD from OD, with the addition of potential adjunctive testing techniques to further define these two disorders (11).

## Risk Factors for Functional Dystonia

FD has several predisposing vulnerabilities, some of which are similar to those of primary OD. FD is more common in females than males (as is typical for FND in general) and has a typical age of onset of 29–50 years, depending on the population tested, compared to a generally childhood, adolescent or early adult-onset in most genetic dystonias (45). Although FD is generally less common in children and the elderly, there is increasing recognition of FD and FMD in general in these populations (45–47). FD is also seemingly more common among white individuals, although this association may be in part related to lack of diversity in research cohorts published to date (45). Of note, this is also frequently the case for OD, where particularly in genetic forms (particularly DYT-TOR1A/DYT1), individuals of Ashkenazi Jewish origin are at higher risk (34, 45). Based on the biopsychosocial formulation, predisposing, precipitating and perpetuating factors are shown in Table 2 (48).

**Table 2:** The biopsychosocial model: predisposing, precipitating, and perpetuating factors for the development and maintenance of functional dystonia and related functional neurological disorders [adapted from McKee et al. (48)].

	**Biological**	**Psychological**	**Psychosocial**
Predisposing vulnerabilities	• Sex—female • Intellectual disability • Comorbid neurological conditions • Other nervous system vulnerabilities • Co-morbid functional somatic disorders (i.e., fibromyalgia, irritable bowel syndrome, other chronic pain disorders) • Sensory processing difficulties sensory processing difficulties	• Comorbid mood and anxiety disorders, PTSD, personality disorders • Dissociation • Alexithymia • Insecure attachment • Temperament and maladaptive personality traits (i.e., obsessive-compulsive, neuroticism)	• Family functioning/childhood neglect • Chronic illness in family • Traumatic death in family • Adverse life experiences (divorce in childhood, home life or personal relationships) • Physical, sexual, or emotional abuse • Financial difficulties • Inadequate social supports • Attitudes toward health and disease • Symptom modeling for dystonia/abnormal movement (through family history, as patterning)
Precipitating factors	• Abnormal physiological event(s), such as sleep deprivation, sleep paralysis, hypnic jerks, hyperventilation, palpitations • Physical precipitating event (acute pain; peripheral limb injury or head trauma; dizziness caused by vestibular event; surgical intervention) • Initial motor compensation following an injury (such as to avoid pain)	• Emotional reactions to physical injury or other life events (sudden loss of loved one, sudden change in social or financial situation, relationship breakdown etc.) • PTSD trigger or flashback • Perception of event as traumatic/negative • Dissociative event • Panic attack (including dizziness as part of panic)	• Loss of employment or other occupational difficulty • Divorce or marital strain • Traumatic death of loved one • Other relational stress
Perpetuating factors	• Physiological arousal • Chronic pain • Chronic fatigue • Abnormal motor habit formation • Deconditioning • Other medical/neurological comorbidities limiting treatment participation • Fixed posturing leading to contractures • Risk if motor pattern is continuous (particularly in fixed posture), this may lead to irreversible changes in the motor program and lack of reversibility	• Negative expectation bias • Negative attentional bias • Illness beliefs including perception of symptom irreversibility or attribution to another cause (of the patient or significant others) • Fear of falling • “No pain no gain” philosophy to healing • Avoidance of symptom exacerbation • Hypervigilance and dissociation • Lack of acceptance of functional neurological disorder formulation • Not feeling believed • Maladaptive behaviors (reliance on walking aids/wheelchair etc.) • Identity linked to rigid concepts around productivity, self-efficacy	• Provider diagnostic uncertainty (ambiguous diagnosis and ongoing investigations) • Social benefits of being ill (often out of awareness) • Reliance on care and disability benefits • Pending litigation and compensation claims • Workmen's compensation/disability • Poor care coordination • Poor family buy in/support of diagnosis and treatment plan • Employer or patient urgency to return to work • Ongoing social difficulties (relationship, financial, loss of roles etc.) • Social stigma around functional neurological disorder • Role in other disorder support groups

*Note, the above list is not exhaustive but rather is representative of the commonly encountered factors that are relevant to consider in developing a patient-oriented biopsychosocial formulation. Also, a given factor may relate to multiple categories, such as alexithymia could be both a predisposing vulnerability and a perpetuating factor. PTSD, post-traumatic stress disorder*.

Family history also plays a role in risk for FD, and FMD more broadly, as well as OD. Despite this, although patients with FD commonly have no family history of dystonia or other movement disorders (34), there is influence of a family history of neurologic and psychiatric disorders on an individual's risk for developing FD (49). A family history of movement disorders has also been shown to increase risk, through potentially patterning motor behaviors, and there have been reports of familial FMD (49). OD may be idiopathic and several genetic forms have been described, with considerable clinical heterogeneity in patients with a certain gene [see Table 1; (50)]. Patients with *DYT1*, the most common genetic dystonia, may also not necessarily have a family history, as there is reduced penetrance in roughly 30%, with frequent non-manifesting carriers (49, 51). Different genetic movement disorders phenotypes are also differentially impacted by genotype. For instance, primary paroxysmal dyskinesias associated with the *PRRT2, GLUT-1*, and *MR-1* genes are associated with distinct presentations (paroxysmal kinesogenic dystonia, paroxysmal non-kinesogenic dystonia and paroxysmal exercise-induced dyskinesia, respectively) and respond to particular treatments (52). However, over time, these diagnostic silos are becoming less robust, with increasing clinical and genetic heterogeneity (53, 54).

Psychiatric symptoms are also frequently associated with FD, with anxiety and depression being the most common psychiatric comorbidities (46). Furthermore, in a study comparing FD and OD patients, a major depressive episode occurring prior to dystonia onset was a significant predictor of a FD diagnosis (55). However, it is notable that anxiety, depression and suicidality are also more common in OD than in the general population (56). Personality disorders have also been reported to be more common in FD vs. OD (55, 57). There is evidence to support that patients with FD also tend to have a more stressful social environment than OD and typically display less extroversion or openness (55).

## Diagnosis of Functional Dystonia

Diagnostic criteria for FD and other FMD has undergone several iterations in recent decades (58). The first official criteria established by Fahn and Williams in 1988 were only for FD (39), and included four diagnostic categories: documented, clinically-established, probable, and possible FD [Table 3; (16, 58)]. In the documented and clinically-established category, incongruence or inconsistency of movements with classical OD were required to make a diagnosis (39, 40). Other clinically supportive criteria included the presence of other functional neurological signs, other somatic symptoms (somatization), or “obvious psychiatric disturbance” (39). In a revision in 1995, the documented and clinically-established categories were collapsed to both constitute “clinically definite” FMD (59). Without further evidence of a functional cause, the presence of only incongruence or inconsistency constituted a clinically probable case. The clinically-possible category included only obvious emotional disturbance, the symptom that is now considered least reliable in diagnosing FD out of the original criteria (40).

**Table 3:** Functional dystonia diagnostic criteria [Fahn and Williams (39)].

Documented^#^	**Persistent relief** by: psychotherapy; psychological suggestion including physical therapy, or by administration of placebo; or if witnessed symptom-free when left alone believing to be unobserved^*^
Clinically established^#^	Dystonia is inconsistent over time or incongruent with classical dystonia (cannot move limbs on request or resistance with passive movement and contrast with ability to perform daily tasks) and **at least one** of the following: 1. Other “psychogenic” (functional) neurological signs (give-way weakness, “false” sensory findings etc.) 2. Multiple somatizations 3. Obvious psychiatric disturbance
Probable	1. Dystonic movements inconsistent or incongruent with classical dystonia but no other “psychogenic” (functional) features 2. Dystonic movements are consistent and congruent with organic dystonia but there are additional definite “psychogenic” (functional) neurological signs 3. Dystonic movements are consistent and congruent with organic dystonia but there are multiple somatizations
Possible	Dystonic movements are consistent and congruent for organic dystonia but with obvious emotional disturbance

* *The authors do mention a caveat, that in making a documented “psychogenic” (functional) dystonia diagnosis that there must not simply be improvement under hypnosis or when using sedatives, as they describe that other forms of dystonia can also improve with these. They also describe rare spontaneous remissions in “organic” dystonia (with an example being cervical dystonia) and that otherwise improvement in “organic” dystonia is typically incomplete and temporary, whereas functional dystonia was noted to have traumatic, sudden improvement*.

# *In a 1995 revision, Documented and Clinically established diagnoses were collapsed into a new category “Clinically Definite” (59). Bolding in table is used for emphasis*.

In 2006, Shill and Gerber proposed a new set of criteria for FMD diagnosis, removing the necessity for incongruence/inconsistency, replacing a “necessary and sufficient” classification with a combination of primary symptoms (40). These symptoms, categorized under clinically-definite, included pain, fatigue, exposure to a disease model, and potential for secondary gain, while multiple somatizations and psychiatric disturbance were considered secondary to those criteria (40). Probable and possible categories had fewer of the diagnostically reliable symptoms (58). These criteria have been criticized for their removal of inconsistency and incongruence from the diagnostic criteria, which many neurologists believe are the most critical factors in making an accurate diagnosis, particularly in FD (40).

Gupta and Lang's 2009 FMD criteria removed clinically-probable and possible from the potential diagnosis, leaving just clinically-definite criteria from Fahn and Williams' initial outline (58). Their modifications also included increased emphasis on laboratory-supported diagnosis (particularly with neurophysiology), and generally minimized the importance of emotional disturbance and patient history in the diagnosis (60, 61). The authors believed false positive diagnoses were sometimes related to a patient presenting with a neurological disorder accompanied by additional psychiatric symptoms, but that this did not constitute a FMD (40).

Although the diagnosis of FD can have helpful associated historical features, diagnosis is made primarily from “rule-in” neurological examination features similar to other FMD, which are inconsistent with OD (40, 62). In an international study of neurologists on their FMD diagnostic practices, two thirds of participants reported having access to electrophysiological laboratory testing to help or confirm their diagnosis (63). In some cases, ordering electrophysiological or other neurological tests may be used as a signal to the patient that the physician is taking their condition seriously, although most often tests are only used in unusual cases when the diagnosis is otherwise unclear (63). Most neurologists reported often referring patients to a psychiatrist for further diagnostic consultation, even if a FMD diagnosis had already been made by the neurologist (63). This may lead to potential issues with the psychiatrist challenging the diagnosis, including reports of no clear “psychiatric diagnosis” being found (64).

There are several challenges in diagnosing FMD, and particularly FD. First, OD may be mistaken for other conditions such as tremor or Parkinson's disease (65). In addition, particular ODs commonly misdiagnosed as FD include paroxysmal dystonia/dyskinesia (52, 53), task-specific dystonias (66, 67), dopa-responsive dystonia and other potentially treatable metabolic movement disorders (68), rapid-onset dystonia-parkinsonism (69), and acute drug-induced and tardive dystonias (57). There are also the various non-FND pseudodystonias (e.g., abnormal postures related to atlanto-axial subluxation, compensatory movements after injury, or similarities in presentation in pathological muscle stiffness/myotonia, etc.), which mimic the appearance of dystonia but result from musculoskeletal disease or dysfunction in the sensory, motor or other neurological pathways (70).

In addition, diagnoses can further be confounded by the presence of FD and other functional neurological symptoms presenting in patients with other neurological disorders, such as the presence of FMD in Parkinson's disease (occurring in 1.4–7.5% in Parkinson's disease patients) (71) or functional seizures in the setting of epilepsy (one in five patients with functional seizures also has epileptic seizures) (72). These two concurrent processes require different treatments and their co-occurrence can at times be challenging to manage.

Incorrect diagnoses may lead to unnecessary investigations, increased healthcare costs (73), iatrogenic harm, possible inclusion in inappropriate clinical studies, and poor prognosis (74, 75). Yet, as has been described, the identification of features incongruous and inconsistent with OD are complicated and based on familiarity with complex and atypical dystonia cases, while also relying on clinicians being comfortable with eliciting diagnostic functional neurological examination signs. It is therefore generally recommended that only a specialist with expertise in distinguishing dystonia from other movement disorders, and FD from other neurological disorders, make the diagnosis of FD (40). However, despite this, diagnostic uncertainty even among movement disorders experts is high, particularly for the most challenging cases (41, 42).

## Distinguishing Clinical Features of Functional Dystonia

OD and FD are not mutually exclusive; patients may present with both functional neurological symptoms and other movement disorders, including OD (21, 71, 76). It is therefore important to closely follow patients over time, particularly in more ambiguous cases with less diagnostic certainty for a definite FD, as features may emerge suggesting an additional or alternative diagnosis misconstrued to be a FMD (77). Hence, it is vitally important to use accepted diagnostic signs in the context of pertinent historical features in a skilled provider to properly distinguish FD from OD.

### Historical Clues Favoring a Functional Dystonia Diagnosis

While not diagnostic, a thorough history frequently reveals features distinguishing FD from OD. Rather than a gradual progression of symptoms, as is generally seen in OD, FD patients commonly report a sudden onset of maximal symptom severity and rapid symptomatic progression (78). Furthermore, in FD, symptoms often arise in response to a physical precipitating event or injury but also stressful or traumatic experiences, or a reminder of such events (79). Peripheral trauma can result in a range of neurological disorders, including FMDs, particularly in the setting of innocuous trauma (80, 81). Spontaneous remissions and subsequent recurrences may also occur in FD and while such remission is very rare in OD, this can be seen, predominantly in cervical dystonia and blepharospasm (82).

Childhood maltreatment (including neglect) represent another risk factor for FD. Such sensitive information may only be revealed if there is sufficient rapport with the clinician. However, when speaking sensitively and empathetically, such information may be succinctly explored even on the first visit if sufficient time allows (83). Compared to OD, patients with FD are also more likely to have psychiatric disorders, particularly anxiety and depression (84); distinct personality traits (85) and psychological profiles, including dissociation, alexithymia, and insecure attachment, are also more common in FND (86). FD also tend to have higher levels of somatic symptom disorder and other functional somatic disorders [e.g., fibromyalgia, irritable bowel syndrome etc.; (58, 78, 87)].

### Pain and Functional Dystonia

FD patients commonly experience pain in both affected regions/limbs and in other body areas (15, 88), and the presence of pain is associated with worse outcomes (15, 88, 89). In comparison, pain is less common in OD, other than in cervical dystonia, where it can contribute to significant disability (90, 91).

In Schrag et al.'s large series of fixed dystonia, 20% fulfilled criteria for Complex Regional Pain Syndrome (CRPS) (92). The association between CRPS and different forms of dystonia was first described in 1986 (93, 94). CRPS involves localized pain, including the presence of allodynia, hyperpathia, swelling, sudomotor, and vasomotor changes, as well as autonomic changes involving both skin color and temperature (80, 92). CRPS is differentiated into two types: type 1 (CRPS-1) occurs in the absence of a nerve lesion, and is the subcategory associated with FD, while in type 2, there is a distinct peripheral or more proximal nerve injury (92). Several diagnostic criteria and characteristic attributes of CRPS-1 are similar to that of FMD (92). For example, CRPS-1 may be similarly inconsistent with other pain disorders and symptoms may be distractible (92). Pain may travel between limbs or affected regions, in the absence of a further injury or inciting event. CRPS also tends to occur suddenly, similar to the abrupt onset typical of FD but not OD (80). Not surprisingly, debate over the neurologic and psychiatric roots of CRPS has mirrored that of FD (92). The origins and pathogenesis of CRPS-1 and dystonia, the “causalgia-dystonia” syndrome, are unknown (80). Hypotheses include that abnormal contractions and posturing of FD are conditioned responses to pain, with heightened expectation of pain triggering disordered movements (92). Other hypotheses focus on the origins of motor control and pain within the central nervous system. For example, neuroimaging studies have examined the overlap between pain and motor control regions as potentially significant in facilitating these associations (92). Another hypothesis contends that abnormal sensorimotor integration contributes to peripheral pain and posturing (95). The undetermined pathogenesis of CRPS-1 has led to frequently inadequate treatment, concentrated on pain alleviation rather than a more comprehensive approach (16, 80). The increasing recognition of sensory processing difficulties in motor FND may also be relevant in FD, an area in need of further investigation (96). In extreme cases, the patient may even seek amputation as a means to alleviate their suffering (16, 97). Such a drastic approach has also been reported in fixed FD as a last resort, following inadequate treatment (98). The reason for this tendency to opt for amputation, similar to that seen in CRPS has been postulated to be related to deficits in body schema and may represent a form of body integrity identity disorder (98).

### Clinical Examination Presentation of Functional Dystonia

**Figure 1:**
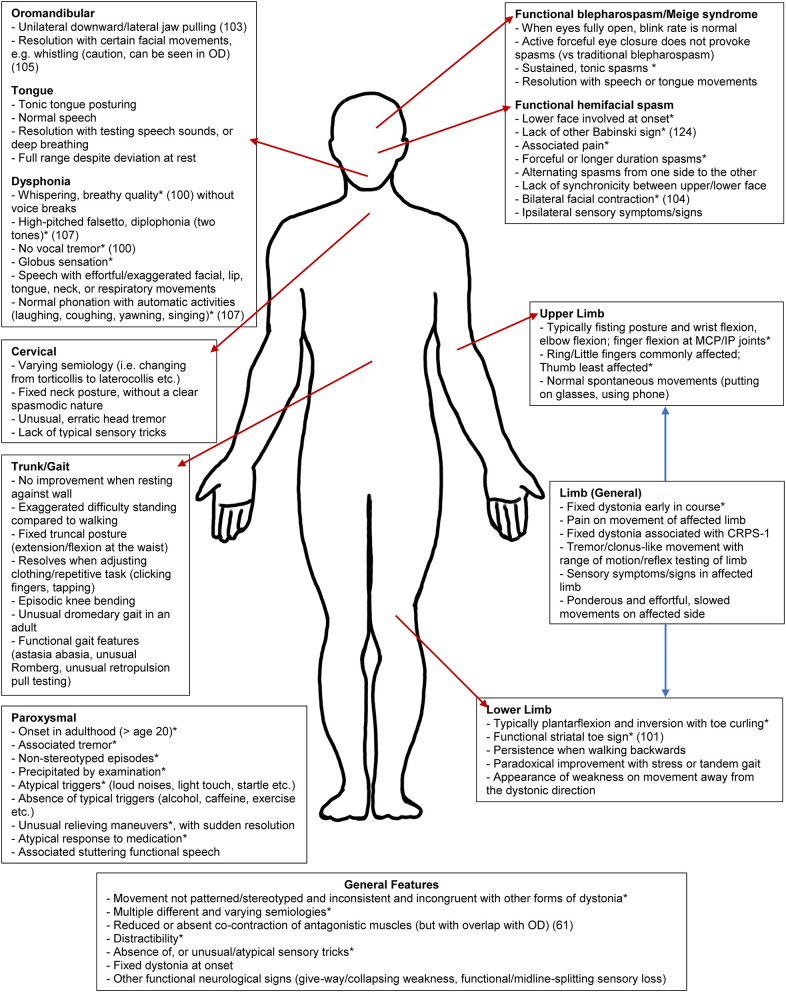
Clinical clues for functional dystonia subtypes. This schematic represents an abbreviated list of potential clinical clues. For the full list, please see Supplementary Table 1. CRPS-1, complex regional pain syndrome type 1. *Previously published (16, 39, 52, 99–107).

The range of published and anecdotal clinical features of FD found by our group are shown in Figure 1 and Supplementary Table 1. A core feature of OD is the internally consistent and stereotyped nature to the movements themselves (although there may clearly be variation depending on certain tasks). In comparison, as a fundamental feature, the nature of FD movements is both incongruous with that seen in OD (such as early fixed posturing, or dystonia initially occurring at rest) but also the movements themselves are highly internally inconsistent and variable, sometimes involving multiple different semiologies of abnormal postures (11). FD symptoms may also greatly fluctuate, such as between taking history and conducting the physical examination (a patient's gait entering the clinic may differ from their atypical gait during examination or marked worsening provoked by video recording) (29, 34, 78). Similarly, symptoms may also appear more frequently during the clinical assessment than the patient reports as typical in their everyday life, owing to this heightened sense of attention, as well as the increased anxiety state associated with the clinical encounter (83). These features are consistent with the role of attention in amplifying functional neurological symptoms more broadly (108).

**Figure 2:**
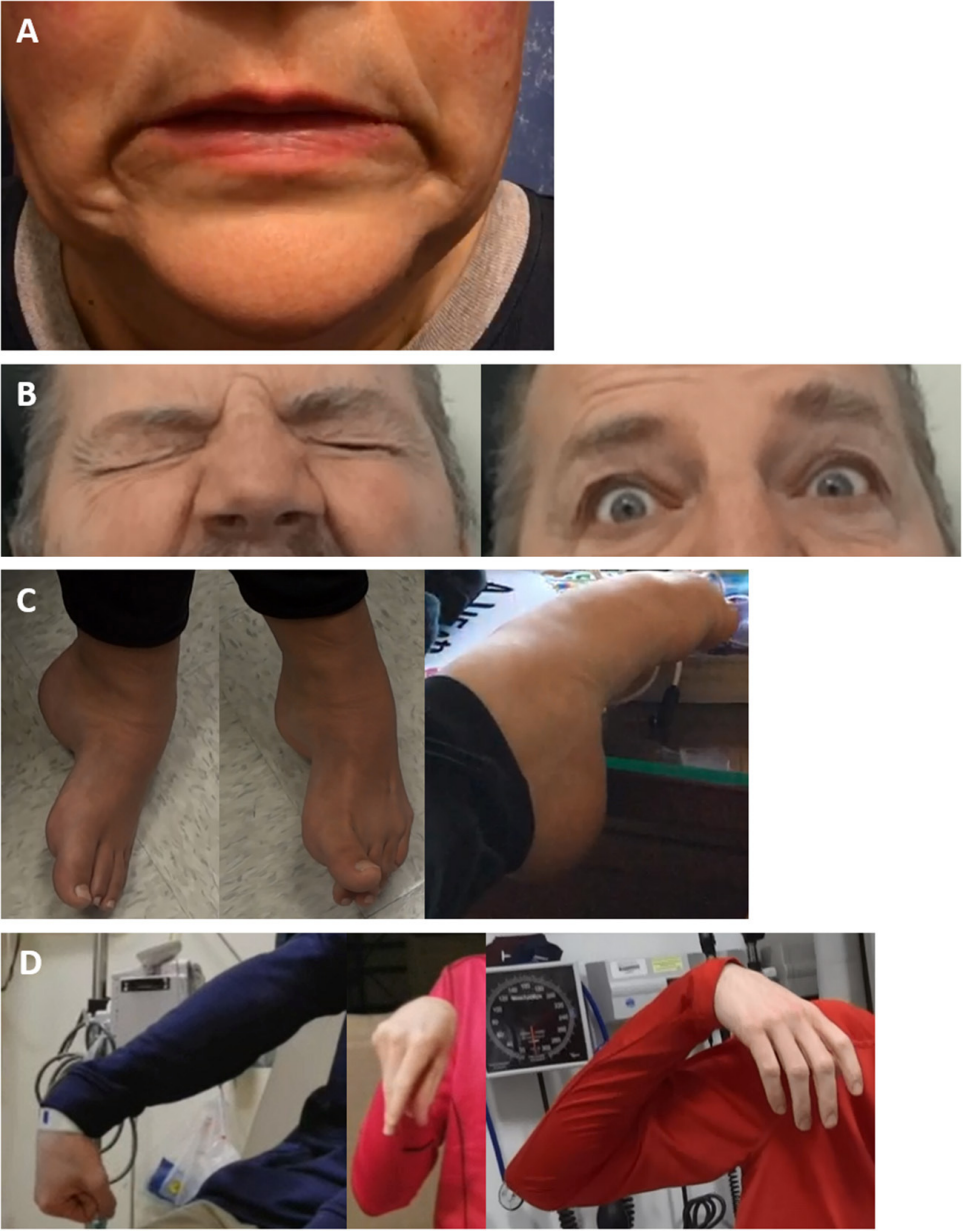
Examples of functional dystonia phenomenology. Examples of functional dystonia phenomenology are shown: **(A)** functional cranial dystonia with bilateral lip pulling; **(B)** functional blepharospasm with eyes tightly shut (left) and forcefully open when concentrating (right); **(C)** three examples of functional foot dystonia, illustrating the typical posturing involving fixed dystonia with plantarflexion, inversion (left), in the same patient with extension of the great toe, with flexion of the others toes (middle), and a different patient with paroxysmal dystonia involving plantarflexion and toe curling (right); **(D)** three examples of varying dystonic upper extremity posturing in a patient with paroxysmal functional dystonia—right arm extended with wrist flexion and fisting (left), elbow flexion with wrist flexion akin to carpopedal spasm (middle), and shoulder abduction, elbow flexion, and wrist flexion with a limp hand (right).

Other general principles of FD can include: (i) lack of a rostrocaudal gradient seen in OD; (ii) rapid spread from a focal to multifocal or generalized distribution; (iii) widespread distribution at onset; (iv) and spread beyond presenting symptoms given that dystonia generally remains focal in adult-onset idiopathic dystonia (39). There may also be inconsistency in distribution over time, with a pattern of distribution sometimes appearing random or cyclical, in contrast to static symptoms or a methodical spread seen in OD (78). If the posturing in FD is not fixed, it may be distractible and suppressed by tasks such as finger-tapping/mental arithmetic, or by suggestion (such as the use of a tuning fork), in comparison to OD (109, 110). As a caution, although bizarre movements and postures are frequent in FD, it is important to acknowledge that unusual posturing, particularly in younger patients, can be a harbinger of rare genetic (2), autoimmune [e.g., NMDA-receptor antibody encephalitis], (111), toxic (112), or metabolic OD (113). This is important to note, as secondary dystonia can potentially be treatable depending on the underlying cause, particularly in metabolic disorders (114). The presence of other FMDs is a hallmark of FD (58). Another important feature which can be present in OD but not FD is the presence of sensory tricks, which are frequently atypical or absent in FD but in comparison, are reasonably common in OD, found in more than half of patients with facial/cervical dystonia (16, 115). Examples of FD clinical phenomenology are shown in Figure 2.

### Clinical Features of Common Functional Dystonia Phenotypes

#### Limb

Abnormal limb posture, particularly involving the hands and feet are common in FD. Patients may experience posturing and contractions of multiple limbs simultaneously, and symptoms may travel across limbs (116). Examples of common functional dystonic lower limb postures include foot plantarflexion and inversion, and first toe extension as other toes flex (58, 99), as illustrated in Figure 2C. In the upper extremities, FD frequently manifests as wrist and finger flexion, sometimes with a clenched fist and generally involving the dominant side (78, 117). If there is more proximal involvement, combined wrist and elbow flexion are typical (58, 99), as shown in Figure 2D. FD posturing is more often fixed than in OD, where symptoms are usually mobile, dynamic and may be task-specific (83). In particular, fixed dystonia at onset is a common FD presentation but highly atypical for OD, where symptoms may become gradually more fixed over time, related to progressively more severe and persistent posturing, although there is still generally an additional spasmodic, dynamic element (12). In FD, limb dystonia frequently starts in the foot, however adult leg dystonia is rare in OD, and uncommon in patients over 30, typically affecting children (12, 88, 100). However, focal foot dystonia may occur as a secondary dystonia, such as in Parkinson's disease, particularly in young onset cases (118). In FD, fixed posturing of the lower limbs causes the patient to drag the affected leg (sometimes behind the patient, as a dragging monoplegic gait), which is persistent throughout the gait cycle. Fixed lower limb FD can be debilitating, may result in loss of ambulation and is frequently accompanied by pain on attempted movement of the affected limb, and in some cases, by CRPS-1 (58, 99, 119). Other common causes of fixed OD include the dystonia seen in corticobasal syndrome, which typically starts on the left (generally non-dominant) side (120). Another peculiarity is that there is no improvement of limb posturing with sleep in FD, whereas in OD, symptoms often briefly improve with sleep (88). Limb FD also may respond to placebo, either with medications or botulinum toxin injections (121). In addition, the use of general or local anesthesia may reduce symptom severity or relax fixed posturing, as well as aiding in the determination of the presence of contractures (122). If fixed posturing is not reverted, contractures may develop, which may mimic OD (122).

In FD, when patients attempt voluntary movement to command in a direction opposite that of the dystonic posturing, this may activate antagonist muscles but with limited (and in some cases apparently no) activity in the agonist muscles, which is very unusual, as co-contraction of antagonistic muscles is a hallmark of OD (61). There can also be an associated functional “striatal toe sign” in FD, where in the setting of fixed great toe extension, there is pain and variable resistance to passive flexion, whereas forced passive dorsiflexion of the second to fifth toes causes spontaneous plantarflexion of the great toe. This is in sharp contrast to an “organic” striatal toe, where there is no pain or resistance on passive flexion of the great toe and dorsiflexion of the other toes does not change the great toe posture (101). Other discriminating examination features include abilities inconsistent with the degree of abnormal posturing, active resistance with passive movement (sometimes despite the appearance of complete immobility), persistent abnormal posturing when walking backwards (which typically improves lower limb OD), or the presence of functional weakness or sensory symptoms (100). Movement of the affected limb may also be inappropriately slow and ponderous but without triggering worse posturing, as frequently seen in OD. Straightening out the FD posture may in rare cases result in a new resting dystonic posture, or shifting from one posture to another, as per the “whack-a-mole” sign (102). None of these features are found in OD.

#### Cranial—Facial Spasm and Blepharospasm

Encompassing a variety of phenotypes, functional cranial dystonia encompasses 16% of all FMD (16). Symptoms may be paroxysmal or persistent, with occasional fixed posturing and may affect several facial regions (16, 103). Like other FD, the initial onset of functional cranial dystonia and pattern of subsequent symptoms are abrupt and seemingly random (103).

In functional facial spasms, this tends to involve the lower face (sometimes at onset) (103, 104), with the most common phenotype (in 84% of patients) being asymmetric tonic jaw/lip deviation (58, 104). An excellent reference is the paper by Fasano et al., who comprehensively reviewed findings comparing functional facial spasm to other forms of facial spasm (103). In comparison to sensory tricks being relatively common in OD, they appeared rare in FD. Speech is typically normal in OD with facial spasms/Meige syndrome (unless there is additional segmental involvement of the vocal cords or oromandibular dystonia), whereas slurred or hesitant speech is not infrequent in FD. Activation of accessory neck muscles with platysma involvement is rare in OD and when it does occur, is bilateral but is common and ipsilateral in FD. The spasms may be induced by action in OD, whereas they are frequently paroxysmal in FD, with episodes of normal facial movement in between. The spread and progression of OD involving the face is generally segmental, with spread to the neck, whereas FD is typified by an unusual and rapid spread to multifocal or more widespread dystonia distribution (103).

The facial pulling may be synchronous or asynchronous and there may be tonic deviation, which is not seen in OD (103, 104). An example of bilateral lower facial pulling is shown in Figure 2A. While the lower face is most commonly affected, other functional cranial phenotypes include the eyes, upper face (forehead and perinasal regions), hemifacial, and rare tongue involvement (16, 58, 103, 104, 123). Tan et al. in their case series reported the absence of spasms during sleep, lack of worsening during voluntary facial movement and bilateral facial contractions, with asynchronous contractions, and occasional associated facial pain (104). Other features include alternating spasms from one side of the face to the other and the presence of very forceful spasms, lasting longer than typical hemifacial spasm contractions (100). We have also observed frequent functional sensory loss on the affected side. Another defining feature is the absence of ipsilateral eyebrow elevation (the “other” Babinski sign), typical of hemifacial spasm (16, 124).

Functional blepharospasm is a rare entity, seen in 0.3–7% of FMD (59, 76) but is overall not an infrequent cause of blepharospasm, accounting for 20% of a single-center series (125, 126). Other unusual features seen in functional blepharospasm, include the presence of unusual visual symptoms and the sudden onset of prolonged spasms, whereas in other forms of blepharospasm, there is increased eyeblink rate slowly evolving into more prolonged spasms (126). An example of a patient with frequent, forceful eyelid spasm, which completely resolves when concentrating and engaged is seen in Figure 2B.

Botulinum toxin injections, with or without facial physical therapy (PT), are the treatment of choice for cranial OD. In FD, patients may have an immediate response to botulinum toxin injections, distinct from the delayed mechanism of action of the toxin (16, 127). On the other hand, cranial OD may have gestes antagonistes, whereas these are frequently absent or highly atypical in cranial FD, and in comparison to improvement of OD symptoms touching the face, in FD, symptoms usually worsen in response to touch (58, 115). Furthermore, in comparison to structural forms of hemifacial spasm, in functional spasm, there is no evidence of vertebrobasilar dolichoectasia on magnetic resonance angiography (104).

#### Cranial—Oromandibular/Tongue Dystonia

Oromandibular dystonia is a rare focal dystonia involving abnormal involuntary contractions of the jaw, lower face, and tongue and may interfere with speech and swallowing (128). Jaw dystonia can be either predominantly jaw opening, closing, or lateral deviation and is associated with muscular tension, pain, spasms, and fatigue in the muscles of mastication and may present initially as bruxism (129). Defazio et al. produced a set of expert diagnostic recommendations, with core features including that movements are patterned and repetitive and are either spontaneous or triggered by certain motor tasks, with or without associated tremor (130). In OD, jaw spasms are typically bilateral (131). Talking and chewing typically worsen oromandibular dystonia (129), however, in some cases there can be a paradoxical improvement when talking or with adopting certain tongue positions (105). In contrast, FD of the jaw typically involves ipsilateral downward and lateral jaw pulling and other associated signs including a fixed posture, ability to voluntarily suppress the jaw movements/posture and the presence of distractibility (103, 130). In comparison, unilateral jaw dystonia is markedly uncommon in OD (131). Oromandibular FD can migrate to other non-adjacent sites or change in nature, such as jaw movements with both opening and closing features that wax and wane. Typically, OD and lingual dystonias, like most head and neck dystonias, preserve vegetative tasks such as swallowing and breathing, and a modified barium or video swallow examination is helpful to document normal oropharyngeal swallowing function. If pain is the predominant complaint, an oral surgery consultation for evaluation for temporomandibular disorders is helpful.

Tongue involvement is rare in OD (generally seen as part of an oromandibular dystonia) and in FMD, is infrequently seen in isolation, and is often associated with other body involvement (126). Functional tongue dystonia is frequently a tonic contraction and may commonly be associated with functional tongue/facial weakness. In FD, there can be “wrong-way” tongue deviation, with deviation toward the side of apparent facial weakness (132). There may also commonly be functional dyskinesias associated with the abnormal posture. A clue toward a functional neurological diagnosis is the presence of normal speech (speech should be dysarthric in the setting of significant tongue dystonia), or stuttering speech (which would be entirely inconsistent). Other indicators are resolution of tongue involvement with testing individual speech sounds (lingual, labial, and guttural) and holding prolonged vowel sounds (“Ahh,” “Eee”), or even deep breathing, similar to that seen in functional palatal tremor (120). The presence of full range and normal active tongue movements despite deviation at rest would also be incompatible with what would be seen in OD, as such movements would generally trigger worsening dystonic movements. We have also seen a case of functional tongue dystonia/dyskinesia where the tongue movements entrained to the direction of gaze when testing eye movements, which would be incompatible with OD. Functional tongue dystonia may respond to similar treatment involving retraining as functional palatal tremor (133).

Suspected oromandibular and tongue dystonias should be seen by an otolaryngologist. The workup and treatment strategies for both FD and oromandibular OD include speech and voice therapy, PT, behavioral strategies, and treatment for temporomandibular joint disease including bite guards and a soft diet. Botulinum toxin for jaw closing muscle groups, such as the masseters, temporalis and pterygoids can have a positive response, depending on the pattern of spasms. Injections to the tongue, submandibular regions and anterior neck muscles are associated with higher rates of swallowing side effects. Response to therapy often helps differentiate FD from OD, including appropriate response to botulinum toxin injections and medications. Other strategies include muscle massage and a soft diet, although being mindful of a view to transitioning over time to a normal diet. Particularly in FD involving the tongue, a video swallow evaluation may provide important information regarding strategies to help prevent aspiration, regardless of the cause, while speech therapy techniques involving retraining of the tongue are important.

#### Functional Dysphonia Mimicking Spasmodic Dysphonia

Spasmodic dysphonia (SD) is a focal primary dystonia involving the laryngeal muscles which affect voice production. The condition is more common in women and the vast majority are of the adductor type, with a small proportion being of the abductor type. The spasms may be accompanied by a dystonic tremor in 20% of cases, and respiratory involvement is rare but has been described with adductor spasms occurring with breathing (134). SD rarely can be task-specific, such as in singer's dystonia (135). Vegetative laryngeal tasks (such as breathing and swallowing) are normal, with the primary symptoms involving voice production, that often worsens with stress, phone use, vocal projection, and public speaking. Sensory tricks can involve touching the nose or chin, humming, laughing, singing, or normal emotional laughing/crying (134). Adductor SD is characterized by vocal strain, with roughness, voice breaks that sound and feel like being strangled, and resulting difficulties with pitch regulation. These voice changes are exacerbated during adductor laryngeal tasks such as counting from 80 to 90. Abductor SD results in involuntary spasms of the muscles that pull open the larynx with a voice quality that sounds breathy, weak, and asthenic (134). Abductor laryngeal tasks such as counting from 60 to 70 will worsen these breathy breaks.

It is difficult to distinguish SD from functional dysphonia mimicking SD. Therefore, patients should ideally be evaluated by a skilled otolaryngologist and voice therapist with experience in the assessment of functional voice disorders and SD, for laryngoscopic evaluation. However, there are several clinical clues, which can be useful for the practicing neurologist (100, 106). Baker provides an excellent review on features differentiating purely functional speech, muscle tension dysphonia (MTD) and other neurological causes of speech disorders (107). In comparison to the rarity of abductor SD, functional SD-like speech is frequently sudden in onset and typically a breathy whisper, while some patients can present with complete aphonia (100). Speech can be either high-pitched (Minnie Mouse-esque), or low pitched and hoarse, there may be occasional diplophonia (two vocal tones), and patients may activate accessory facial, tongue and chest wall movements in the struggling production of effortful speech (107). In FD, vocal tremor is rare and is not associated with speech arrest (100). The characteristic vocal cadence and rhythm for SD that involves the sudden and intermittent, involuntary spasms during adduction and abduction tasks are not consistently seen in FD, where speech can be markedly variable. FD patients frequently describe an associated globus sensation, which is generally not reported in SD (107). Another hallmark, is that normal or greatly improved phonation may generally not be produced voluntarily but can be unconsciously produced, either during reflex activities (coughing, laughing, yawning or with excited/angry interjections), with other tasks, such as counting, reciting, or singing, or with distraction facilitated by the assessor, such as when performing a cognitive task in functional voice disorders (107). In comparison, normal speech is not able to be produced in SD during these tasks. Normal resonance in functional dysphonia may also sometimes be produced when the patient is asked to count as far as they can in a single breath, generally on reaching the end of the breath.

The neck examination in functional dysphonia is often notable for discomfort while palpating the thyrohyoid space and cricothyroid space. In SD, the larynx is elevated and retracted, and sometimes the thyroid notch is directly adjacent to the hyoid bone, resulting in significant discomfort with manual manipulation of the thyrohyoid space. An experienced voice therapist can often differentiate between FD and SD based on the clinical response from appropriate techniques directed at relaxing and unloading laryngeal tension. In SD, laryngeal examination reveals hyperkinetic activity, with excessive, exaggerated, and involuntary vocal contractions, in either adductor or abductor direction with voice production, but normal bilateral motion on most other laryngeal tasks. Functional MTD laryngeal patterns include sustained lateral or anterior-posterior hyperfunction of the larynx, without the spasms, or severe hyperfunction at the anterior vocal folds and false vocal folds, but with a persistent open glottis. MTD can present with multiple laryngoscopic voicing patterns that demonstrate vocal strain and uniform hyperadduction of the vocal folds and supraglottis across voice tasks (136). Further laryngoscopic characteristics suggestive of a functional cause include the lack of a vocal tremor, no phonetic variability, worsening at the end of a breath, constant false vocal fold constriction and normal laryngeal structure and function (106).

The treatment of functional dysphonia is centered around voice therapy. Therapy techniques involve reducing vocal strain, improving efficiency, and relaxing the laryngeal muscles. Functional dysphonia can have a variety of presentations and it takes a skilled and experienced voice therapist with persistence and a large “toolbox” of techniques for success. Adjuvant medical treatments for functional dysphonia can include sensory nerve blocks, if there is a significant pain component to the spasms, and botulinum toxin injections to weaken and rebalance muscle groups. While botulinum toxin injections can help alleviate vocal strain, the goal is to enable and improve voice therapy techniques. Manual massage and masking can also be useful.

#### Cervical

Functional cervical dystonia often develops in response to trauma or injury of the head, neck, or shoulder regions (58, 94). This form of FD generally occurs rapidly, is frequently painful and immobilizing and may be associated with other abnormal posturing in the body but cannot be explained by a specific nerve injury (137). The most common form is post-traumatic painful torticollis, frequently involving laterocollis and ipsilateral shoulder elevation with contralateral shoulder depression (58). In cervical FD, neck posturing may be frequently fixed, which is highly atypical for OD but can be seen in neuroleptic-induced cervical dystonia, where fixed extension (retrocollis) or flexion (anterocollis) may occur (138). Another feature seen in cervical OD is the “shirt collar sign,” where the shirt collar tends to be shifted to one side (which appears related to the presence of a cervical shift in the photographs presented by the authors), whereas the shirt collar appears straight in FD (139). Cervical FD can also be distinguished from OD by its frequent association with pain, often out of proportion to the degree of posturing, lack of effective sensory tricks (which are more common in cervical than other forms of OD) and other comorbid functional neurological symptoms (58, 137). Treatment of both OD and FD can involve targeted PT, while leveraging sensory tricks and botulinum toxin injections are the mainstay of therapy in OD. It is notable that targeted botulinum toxin injections in some FD patients may yield benefit, particularly if there is considerable objective muscle spasm and abnormal posture (58). In comparison to FD, DBS can be used in cases of pharmacologically refractory OD (140).

#### Paroxysmal

Paroxysmal OD typically arises early in life generally in childhood or adolescence (141–143) and individuals presenting with paroxysmal dystonia/dyskinesia above the age of 21 should broaden the differential diagnosis and clinicians should consider a FMD as a possibility. Furthermore, while paroxysmal dyskinesia and dystonia are very rare, these are common phenotypes in FD. Paroxysmal FD is the most common paroxysmal FMD, with paroxysmal events often presenting with more than one form of FMD and with an average age of onset of roughly 38.6 years (52). Paroxysmal FD manifests as frequently sudden episodes or bursts of dystonic-appearing contractions, which can affect virtually any region of the body, including the limbs, trunk or face (16, 144). Functional paroxysmal symptoms can be differentiated from OD by their variability and frequent inconsistency in semiology (16, 52), as shown in Figure 2D. The duration and frequency of episodes often widely fluctuates, as well as the type and nature of the dystonic and/or dyskinetic movements (16, 52, 144), and episodes may be triggered by, or resolve in unusual or medically inexplicable ways (52, 144). In comparison, paroxysmal OD patients tend to have consistent, stereotyped episodes, with similar episode durations. Despite some ambiguity in cases of paroxysmal FD and our general understanding of the disorder, some research suggests that the prognosis for patients is relatively better than other FD phenotypes (52).

## Pediatric Functional Dystonia

Pediatric FD has received relatively little study compared to their adult counterparts, with few published case series, generally in the setting of other FMDs (145). Similar symptoms, risk factors, and patient histories tend to exist between FD and other FMDs (146). Further research into and recognition of pediatric FMDs and FD is especially important, particularly as symptoms may be debilitating and frequently persist into adulthood (147, 148). As in adults, a shorter illness duration is predictive of a better prognosis in FMD, while childhood functional somatic symptoms can be a useful predictor for future mental and physical health issues, including development of FND as an adult (149).

In comparison to adults, FMD in children are felt to be relatively rare (2–5 per 100,000 children) but involve 6–15% of outpatient neurology diagnoses, although some of this may be related to misdiagnosis (147). Studies indicate a mean age of onset of 11–14 years (146, 147, 149) and is less common below the age of 10 (very rare in some series) (146, 148, 150). There have been some cases as young as age 3 reported (151), although there is concern that at this age, the most common cause may be behavioral. Sex is typically female, as is seen in adult FMD, with girls more likely to develop FD, as opposed to other forms of FMD (147). Of note, the proportion of girls increases after adolescence, compared to a more equal sex distribution in younger children (151). Some evidence suggests that after symptom onset, the course tends to be more episodic than is seen in adult patients (146, 151). Compared to adult FMD, children more frequently experience symptoms in their dominant limb, and less frequently have an additional underlying classical movement disorder (110, 147). There have also been episodes of “mass hysteria” with outbreaks of FMD in children patterning the abnormal behaviors of their peers (152).

FD is a common manifestation of pediatric FMD and in some case series, the most common phenotype seen (148). Risk factors for pediatric FMDs are similar to those for adults, such as high levels of environmental or social stress, comorbid psychiatric disorders (anxiety greater than depression), and precipitating traumatic events and injuries (145, 147, 148, 153). In children, trauma and stress typically manifest in the form of familial stressors, including domestic violence and physical abuse, separation from attachment figures, health issues in the child or a family member, the presence of a parent with a psychiatric disorder (146, 147) and have low rates of sexual abuse (although this may be related to reporting error) (145, 151). Precipitating factors in FD often involve a minor injury (of which falls, a sting from an insect, jellyfish or an animal bite have been described), in addition to a surgery or other medical procedure (148). Other factors more exclusive to children include recent vaccination, problems at school (including difficulties with school work, bullying, or absenteeism), as well as participation in competitive activities such as sports or ballet (145, 151). Disturbances during infancy, including trouble feeding, sleeping, or reacting, may also signal future risk for functional somatic symptoms later in childhood (149). Eating disorders have also been reported to complicate pediatric fixed FD (154).

Pediatric FD often presents with fixed posturing (15, 147, 148, 154), and is frequently associated with pain in the affected limb (15, 146, 154), or with sudomotor changes consistent with CRPS-1 (154). As in adults, prolonged abnormal posture can lead to contractures (122). Treating pediatric FD patients must be handled delicately, as social factors contributing to the disorder may be complex and sensitive for the child, and treatments must be age-appropriate (15). Ideally, treatment is multi-disciplinary and this can involving neurology, psychiatry, relaxation techniques and breathing exercises, cognitive behavioral therapy and tailored rehabilitation, including Mind-Body programs (15, 145). Hypnosis and meditation may also be used to relax postures and alleviate stress (15, 149). Pediatric FD and FMD symptoms have sometimes resulted in unnecessary surgeries, procedures and medication treatment (147), as well as orthopedic surgeries to correct fixed posturing (154). Owing to the frequently high severity of symptoms, FMDs frequently impact academic performance at school and can be associated with school absences, requiring home schooling in up to 50% (147, 148). Although a “diagnostic odyssey” is common in FMD in general, this is especially the case in pediatric FD, where most patients are extensively investigated (148, 151).

## Adjunctive Diagnostic Testing in Functional Dystonia

Study of EMG has been utilized in research but is not yet used clinically in the assessment of FD. In a study using reaction time and co-contraction to attempt to differentiate functional FD from OD, although FD patients tended to have longer reaction times and lower levels of co-contraction during voluntary movements, there was overlap between the two groups, suggesting that even if these two parameters could be useful in differentiating at a group level, they were not suitable for diagnosis at the individual participant level (155). EMG has also been advocated in pediatric FMD, with electrophysiology being a supportive criterion of a functional origin in 4/5 patients with mixed FMD (146). Surface EMG can be a useful adjunct to clinical examination (for instance electrophysiology demonstrating distraction or entrainment) with some caution in the setting of FD, given the overlap with OD. Using brainstem evoked potentials, investigators compared the R2 blink reflex in patients with atypical (presumed functional) blepharospasm and other forms of blepharospasm and found that the R2 recovery cycle was normal in functional blepharospasm but abnormal in 9/10 of the other blepharospasm cases (156). This is an interesting research observation but there is insufficient evidence to recommend this clinically.

Other adjunctive maneuvers and strategies to contribute to the clinical examination have been suggested. These include the placebo immediate response to botulinum toxin injections in fixed and other forms of FD (123) and the role of general anesthesia (157), which can be useful in the diagnosis of complex cases. The “swivel chair test,” where a patient propels himself forwards and backwards on a swivel chair, was normal in comparison to clearly impaired gait in 8 of 9 functional gait patients (which may be frequently present in FD) but similarly affected in other gait disorders (158). They did caution that in patients with generalized dystonia, there may be a false positive chair test, as this can serve as a geste antagoniste, resulting in improvement, and as another limitation, there was no blinding of the movement disorders neurologist performing the test (158).

Transcranial magnetic stimulation (TMS) has been investigated to distinguish FD from OD. Reduced intracortical inhibition and cutaneous silent period have been demonstrated in both FD and OD (95, 159, 160), while in one study, forearm spinal reciprocal inhibition was reduced only in FD (159). However, there have been discrepancies in assessments of cortical plasticity, which was noted to be abnormal only in OD in one study (160), and in patients with CRPS-1, there was also normal sensorimotor plasticity compared to controls. However, cortical plasticity was abnormal bilaterally in both OD and FD in another study, suggesting possible limitations and less separation between diagnoses (161). In this study, the presence of bilateral involvement despite unilateral dystonia may suggest a trait/endophenotype rather than a consequence of abnormal muscle contractions (161). The use of TMS in dystonia is currently of research interest but could have future clinical implications.

In comparison, intraoperative physiological recordings during both thalamic and globus pallidus internus (GPi) DBS surgery erroneously performed on patients with FD, firing rates and thalamic reorganization were similar, while EMG coherence and thalamic signal-to-noise ratio were different in FD and OD, suggesting similarities and differences between these disorders (162). However, in another study involving the GPi, neurophysiology measures did not differentiate these disorders (163).

“Laboratory supported criteria” for other FMD has been suggested, and in areas with significant experience in the evaluation of these disorders, may greatly add to helping verify the diagnosis, particularly in challenging cases and hence reduce unnecessary delay in diagnosis and subsequent unnecessary workup (156, 164). However, there are no straightforward tools to apply to FD, and FD and OD share several neurophysiological features, suggesting some associated pathophysiology. To date, there are no or reliable discriminators for these disorders.

## Imaging and the Pathophysiology of Functional Dystonia

Standard clinical imaging has been used to aid in differentiating FMD from some other movement disorders. Excluding a brain or spine lesion is performed when indicated by history, physical examination, and investigations to rule out structural pathology. In general, dystonia has normal imaging, although certain forms of primary dystonia may show brain degeneration and secondary dystonias can also have various abnormal and sometimes pathognomonic imaging features [e.g., the eye of the tiger” sign in neurodegeneration with brain iron accumulation or the cerebral degeneration seen in X-linked dystonia parkinsonism; (165)]. FD should also have normal imaging, although there is always the possibility of an incidental finding or a neurological comorbidity.

In neuroimaging research, Schrag et al. performed positron emission tomography (PET) in 6 patients with fixed FD, 5 with DYT1 leg dystonia and controls at rest and during an active motor task (166). At rest, while OD had increased blood flow in the left primary motor cortex and thalamus and decreased blood flow in the cerebellum; FD had opposite findings, with increased cerebellum and basal ganglia and decreased primary motor cortex blood flow (166). In comparison, during the movement task, both OD and FD had abnormal activation in the right dorsolateral prefrontal cortex (166). In addition, a resting state study fMRI study in a mixed FMD cohort (*n* = 35; 43% with abnormal posturing) compared to controls revealed decreased functional connectivity between the right temporoparietal junction (TPJ) and sensorimotor cortices after controlling for psychiatric comorbidity (167). The right TPJ is believed to serve as a general comparator of internal predictions/motor intentions with actual motor events, and is implicated in impaired self-agency in FMDs (168).

Several studies have demonstrated abnormalities in brain regions implicated in emotional processing in FD (169). Voon et al. in 16 mixed FMD patients (2/16 with dystonia and 2/16 with multiple phenotypes including dystonia) found increased functional connectivity between the right amygdala and the right supplementary motor area when viewing affectively-valenced faces (170). In another fMRI study of 10 patients with FMD (FD *n* = 2) exposed to emotionally-valenced images, there was increased activity in the cerebellar-limbic network (cerebellar vermis, posterior cingulate cortex, and hippocampus) involved in processing emotional salience (171). Furthermore, Espay et al., in an fMRI study of 12 patients with FD paired with 12 patients with primary OD and 25 healthy controls, probed neural activation profiles using a finger-tapping task (motor task), a basic emotion-recognition task (emotional faces task), and an intense-emotion stimuli task (172). During an emotional faces task, there was decreased right middle temporal gyrus and bilateral precuneus activation and increased activation in several brain regions including the bilateral cerebellum in patients with FD compared to the control groups; during an intense emotion processing task, there was decreased left insula and motor cortex activations in FD compared to OD, along with decreased right opercular and motor cortex activations compared to both comparison groups (172). There were no activation differences across groups in the motor tasks (172). A separate resting state fMRI study of FD (*n* = 40, 12 fixed and 28 mobile FD) revealed among other findings that the entire dystonia group compared to healthy controls showed increased connectivity between the left amygdala and bilateral thalamus; additionally the dystonia cohort showed decreased resting-state functional connectivity between the right TPJ and dorsal and rostral prefrontal regions (173).

In a quantitative structural MRI study of 44 mobile (*n* = 31) and fixed FD (*n* = 13) and 43 age-matched controls, mobile FD revealed volume loss in gray matter structures (nucleus accumbens, putamen, thalamus, caudate nuclei) compared to controls, while compared to fixed FD, mobile FD revealed hippocampal and globus pallidus atrophy (174). Individuals with fixed FD also revealed white matter alterations in the corpus callosum, corticospinal tract, anterior thalamic radiations, and long-range tracts bilaterally compared to controls and mobile FD (174). In a separate study of mixed FMD (35% FD), there was increased volume in limbic circuitry (unilateral amygdala, striatum, cerebellum, fusiform gyrus, and bilateral thalamus) and decreased sensorimotor cortex volume, although these volumetric profiles did not correlate with disease duration or severity (175). These data suggest that FD is associated with structural and functional brain network alterations, although additional research is needed to investigate if these changes are disease-related, compensatory, or related to comorbidities among other possibilities (176).

Altered sensory processing has also been found to be abnormal in FD. As a preliminary observation in 7 patients with fixed dystonia, all noted a subjective abnormal perception of the position of their ankle, feeling as if the ankle was straight with eyes closed but when this was physically straightened by an investigator, felt “abnormal” (119). Temporal discrimination (time interval between two distinct stimuli to be recognized as being separate from each other) has been found to be abnormally prolonged in different forms of OD (177). In a study of mental rotation and temporal discrimination in 11 patients with fixed FD and 11 mobile OD, OD revealed abnormal temporal discrimination and mental rotation, while FD only had abnormal mental rotation (178). It is also notable that in a study of body identity disorder, there was also abnormal rotation perception (179). However, in a study of temporal discrimination threshold comparing FD with OD using paired non-noxious electric shocks, this was abnormally long in both groups compared to controls and there were no significant differences between FD and OD, including when comparing the affected and unaffected limbs (180).

## Management of Functional Dystonia

### Delivering the Diagnosis and Communication in Functional Dystonia

Once the diagnosis of FD has been made, appropriately communicating this to the patient is the first step in treatment (181, 182). Crucial to the treatment of FD is the patient's acceptance and understanding of the diagnosis. Functional disorders in any organ system are frequently met with some level of skepticism and stigma; patients may feel that their diagnosis is “fake” and “all in their heads,” that their complaints are not being met with serious consideration on the part of the physician, or that treatment may be futile (64). The sometimes uncertain and delegitimizing nature of the diagnosis leads patients to “doctor shopping,” as patients seek alternative opinions, not fully accepting their initial diagnosis and as a result postponing appropriate treatment (64). Effective communication while delivering the diagnosis facilitates patient understanding of and receptiveness to their diagnosis (63). To deliver the diagnosis, the clinician should emphasize that FND is real, common, brain-based, and treatable, underscoring that the rationale behind the diagnosis is based on neurological examination findings (12, 182–184). Providing education materials through websites such as www.neurosymptoms.org or www.fndhope.org can be helpful. The physician should also draw direct associations between specific aspects of the treatment plan and the symptoms each attempts to mitigate (185). We generally recommend not making explicit links between FD symptoms and stress (unless the patient is already doing this for themselves), given that while these relationships may be important in some patients, they are often indirect and nuanced (64, 186). The term “functional” is also preferred to “psychogenic” given that functional is an etiologically neutral framing. Similarly, it can also be helpful in early conversations to focus on “what” is the diagnosis (based on examination), while the “why” can be explored by the patient during the course of evidence-based treatment. Prior to discussing a treatment plan with the patient, the evaluating neurologist should determine the patient's overall level of acceptance (buy-in) to the diagnosis. For patients that are very skeptical and seemingly rejecting of the diagnosis, it can often be helpful to ask them to review materials online and to return for a follow up visit to continue the discussion without referring to specialized treatment (as doubts around the diagnosis are likely to limit treatment engagement). Another issue that can be a barrier to making the diagnosis and correctly and sensitively relaying this to the patient is related to the provider's own views on FND, which colors the entire perspective of this condition (187). Fortunately, with greater awareness, understanding and acceptance of FND as a legitimate diagnosis, these views are slowly changing, to the betterment of patients. For those that are receptive to the diagnosis, the sections below discuss available evidence-based treatments.

### Multidisciplinary Treatment of Functional Dystonia

Optimal FMD treatment is multidisciplinary, involving input from neurologists, psychiatrists, physical, occupational, speech and language and psychotherapists, and others, as necessary (183). Medication, including botulinum toxin injections, and pharmacological treatments for underlying psychiatric disorders may also provide some benefit (183–185). In cases where those primary treatment methods do not sufficiently resolve symptoms, other specialized forms of rehabilitation may be considered, including hypnosis, assessment under anesthesia, or electrical nerve stimulation (183, 184).

Physical rehabilitation is vital for the treatment of FD and other FMDs (184). Across several surveys of neurologists, ~80% concurred that PT was a key component in an effective FMD treatment plan (63, 185, 188) and 60–70% of FMD patients experience some improvement of symptoms with PT (184). FD patients require a range of different rehabilitative methods, reinforcing understanding of their diagnosis through exercises and practice. Patients should be given a graded home exercise program, surpassing each stage once physically capable until they reach effectively normal movement (185). Another tenet of therapy involves demonstration of normal movements by the physician or therapist, and positive reinforcement of those normal movements in the patient (184). The distractibility of certain FMDs can also be harnessed to retrain normal movement via simultaneous cognitive or motor tasks, and the role of attention and distraction can be explicitly discussed with the patient as part of the therapeutic process (184). Physical activity and meeting rehabilitation goals has also been shown to have positive psychosocial effects on patients with FMDs, which is particularly significant for patients with poor social or emotional environments or psychiatric illness such as anxiety or depression (189). Furthermore, special attention should be given to teaching awareness of the patient's contractions and posturing so the patient may voluntarily counteract them, such as by resting a limb on a weight-bearing surface (184).

### Physical Therapy for Functional Dystonia

Evidence supporting PT as an integral component of a multimodal treatment approach for functional neurological disorders has amassed over the last half century (185, 190–194). In 2015, a group of clinicians with experience in treating FND developed consensus recommendations to guide PT treatment (184). The authors recommend a program incorporating four principal components: Nielsen et al. (185) education to enhance the patient's understanding of FND, Trieschmann et al. (190) demonstration that normal movements can occur, Ness (191) retraining movements with diverted attention, and Speed (192) changing maladaptive behaviors that provoke symptoms. Additionally, the literature supports treatment progressions that are both individualized and consistent with progressions for similar neurologic conditions. For example, if the patient is having difficulty standing, postural-control training may be initiated in a supported standing position, allowing normal elements of postural control to occur. Positive responses are identified, practiced, and reinforced.

Unfortunately, there is little to no reporting in the literature with respect to specific physical interventions for treatment of FD; this includes that the intensity and duration of physical therapy for FD and related FMDs has yet to be operationalized (195, 196). In treating FD, the therapist uses similar strategies as those used when managing OD, while incorporating principles consistent with the consensus recommendations. Posturing of a limb or body part associated with a focal dystonia may be reduced by changing the nature of the task causing the dystonia or by altering the patient's posture relative to gravity to identify positions in which the patient can begin to move the affected body part (see Table 4). Research in motor learning supports the use of external targets to designate the direction and amplitude of the desired movement (197). The use of mirrors or direct visualization of newly gained positions or movement may help to resolve the discrepancy in perceived vs. actual joint or body part posturing (119). Once positive change in muscle tension or posturing is identified, the patient is encouraged to utilize that position to activate the desired movements and to practice them daily. As the patient demonstrates mastery, the amplitude of the movements is increased, and then the position in which the patient generates the movements is progressed until they can be performed against gravity and within the scope of each desired movement.

**Table 4:** Physical therapy approaches in functional dystonia.

**PT intervention**	**Examples**	**Rationale**
Distraction strategies	- Counting steps out loud while walking - Counting backwards by 3's - Naming fruits that begin with sequential letters of the alphabet	Distraction reduces the focus of the central nervous system on the altered posture/movement
Change posture/position	- LE - Supine, Prone knee extended, Prone knee flexed -Cervical - Supine, Prone, Semi-recumbent	Changing the body or limb posture relative to gravity alters sensory input and can minimize abnormal posturing allowing for active movement
Weight bearing through extremity	- Sitting with foot on an incline in some plantar flexion - Sitting with foot on a ball - Standing with UE or trunk support	Changing the sensory input of the extremity can alter the motor output
Graded task performance	- Begin with whatever movement the individual can perform actively, opposite the preferred posture	Begin slowly with goals for performance set in conjunction with the patient to promote intrinsic motivation. Gradually incorporate segmental limb movements then full body using functional tasks
Elicit automatic movements	- Generate postural control reactions in sitting and standing that engage head and neck for cervical dystonia and the LE for LE dystonia - Walking quickly, walking backwards or running may reduce altered posturing	Reflexive and automatic movements are often retained and can be used to demonstrate to the individual that typical movement is available. Increasing speed of movement might reduce posturing
Sensory - motor retraining	- Direct visualization of normalized movement - Use of mirrors for visualization of improved body posture	Actual vs. perceived body/limb/joint posture might be disparate. Visualization helps reduce the disparity
Interventions to avoid	- Passive stretching	Attempting to alter the posturing passively may result in increased muscle contraction of already overactive muscles
	- Orthotic devices/adaptive equipment	The goal is to reduce attention to the altered posture or movement
Additional considerations	- Promote a schedule of daily activity. Start at a level the individual can achieve and increase slowly every few days	Altered postures and difficulty moving can result in reduced activity levels

*UE, upper extremity; LE, lower extremity*.

The use of orthotic devices and/or adaptive equipment is discouraged, as it brings unwanted attention to the involved body part or dysfunction. Splinting or bracing may be used to prevent joint injury or damage but should be done so judiciously, and weaned as quickly as possible. Likewise, the use of canes, walkers, or other adaptive equipment should be used to promote ambulatory function (for example—to facilitate a safe discharge from the hospital) but removed once safe ambulation has been restored. Examples of PT approaches and their rationale are shown in Table 4.

### Occupational Therapy for Functional Dystonia

Occupational therapists (OTs) have been identified as an integral member of the multidisciplinary team in the care of patients with FND (198), including FD (15). Given training in mental health and expertise in using meaningful activities to engage patients and increase participation, OTs are well-aligned to aide these patients in managing symptoms, developing coping strategies, changing behaviors, and improving function. Until recently, there has been little in the literature regarding OT assessment and treatment to guide clinicians in the treatment of FND and FD. Nicholson et al. recently published consensus guidelines for the occupational therapy assessment and treatment of FND (199). Interventions used with patients who have FD are provided within these guidelines, including the following: encouragement of normal posture and movement through use of distractions (i.e., physical and cognitive), eliciting automatic movement (i.e., weight bear in quadruped, ball toss, etc.), changing posture/positions while performing an activity (i.e., work within gravity eliminated positions, avoiding prolonged postures at end ranges of motion), and developing and implementing a balanced daily schedule to ensure graded re-introduction into daily living skills (199). Splinting should be used cautiously (nighttime splint only) as to reduce risk for non-use of the limb.

Outpatient OTs at MGH have experienced some success in the treatment of FD using guidance from current literature. The treatment approach uses a combination of interventions based on FND guidelines to treatment such as diverting attention to normalize movement patterns (184, 199), in conjunction with interventions for focal OD (200, 201). These can include motor retraining through use of mental imagery, visualization and mirror therapy and sensory retraining, by having the patient palpate and identify raised letters of the alphabet, find coins in a container of rice, or put shapes into matching holes. These retraining techniques can also be considered as use of distraction exercise to enhance the sensory and motor systems for re-education and encourage normal movement patterns. Examples of OT strategies are found in Table 5.

**Table 5:** Occupational therapy approaches in functional dystonia.

**OT Intervention**	**Examples**	**Rationale**
Distraction strategies	- Promote normal movement through use of physical (i.e., manipulate item in hand) and cognitive (i.e., counting backwards from 100 by 3) strategies	Distraction reduces the focus on the altered posture/movement
Change posture/position	- Use gravity eliminated positions to achieve improved performance with functional tasks and posture at rest - Avoid prolonged postures at end ranges of motion	Changing the body or limb posture relative to gravity alters sensory input and can minimize abnormal posturing allowing for active movement
Graded task performance	- Focus on reintegration of affected limb with activity-based functional tasks	Begin slowly with goals for performance set in conjunction with the patient to promote intrinsic motivation. Gradually incorporate segmental limb movements then full body using functional tasks
Elicit automatic movement	- Ball toss, weight bearing in quadruped, sit on therapy ball	Reflexive and automatic movements are often retained and can be used to demonstrate to the individual that typical movement is available. Increasing speed of movement might reduce posturing
Motor retraining	- Mirror therapy - Visualization - Mental Imagery	Actual vs. perceived body/limb/joint posture might be disparate. Visualization helps reduce the disparity
Sensory retraining	With eyes closed: - Palpate and identify raised letters of alphabet - Use limb to search for coins in rice - Put shapes into matched holes	Actual vs. perceived body/limb/joint posture might be disparate. Visualization helps reduce the disparity
Coping strategies	- Guided meditation - Progressive muscle relaxation - Diaphragmatic breathing	Patient's ability to handle stress and anxiety and providing technique to allow relaxation of muscles
Sensory modulation training	- Explore and use different sensory tools based on preference (i.e., eating strong tasting mint/candy, weighted blanket, etc.) - Develop and use a Sensory Diet (planned use) to facilitate self-awareness of positive change	To assist with process of regulating specific behavioral responses to sensory stimuli
Interventions to avoid/limit use of	- Splinting to prevent non-use of limb - Adaptive aids - May consider use at rest (i.e., positional splint while sleeping)	Attempting to alter the posturing passively may result in increased muscle contraction of already overactive musclesThe goal is to reduce attention to the altered posture or movement and not provide reliance on aids
Additional considerations	- Develop functional goals vs. symptom-based goals - Encourage normal movements through participation in functional activities	Altered postures and difficulty moving can result in reduced activity levels and to promote normal activity where possible

Patients with FND and FD are often impacted by psychological co-morbidities and pain. Ranford et al. have found that patients with FND often endorse sensory processing difficulties that are commonly seen in patients with anxiety and post-traumatic stress disorder (96, 202). They suggest obtaining a sensory profile of patients with FND to assist with developing interventions. There are multiple approaches to treating sensory modulation dysfunction in the literature. Examples include promoting daily self-regulation through implementation of sensory strategies/experiences (i.e., fidget with stress ball, eat strong tasting mint, use weighted blanket, or pulling a resistance band) and training use of coping strategies such as progressive muscle relaxation, diaphragmatic breathing, and guided meditation (203, 204). Incorporating these sensory-based interventions may enhance patient recovery when provided in combination with other available treatments.

It is important to develop concrete goals and measure progress, as their symptoms may not change, however functionally individuals may make gains. To aid in documenting clinical change, there are several outcome measurement tools which can be found in the OT consensus guidelines (199). Development of an individualized treatment plan based on functional goals may assist to maximize patient outcomes.

### Other Treatment Approaches

Patients with speech or swallowing symptoms may benefit from targeted speech and language pathology input, particularly for oromandibular and other cranial FD, or if these form part of the symptom complex, using approaches similar to that of PT and OT (205). The use of cognitive behavioral therapy (CBT) to examine relationships between FD symptoms, thoughts, behaviors, emotions and life factors is generally also considered an emerging first line treatment for FD and related FMDs (although more research including larger scale randomized controlled trials are needed) (206, 207). Psychotherapy can also help address psychological trauma and other stressful events or environments that may have contributed to the onset of FD (58). The self-guided CBT workbook, “Overcoming Functional Neurological Symptoms: A Five Area Approach,” based on the clinical trial by Sharpe and colleagues, can be a helpful recommendation; in our clinical program, we generally recommend that this workbook can be used as a guide in the course of individual psychotherapy (208). Psychopharmacology may also be beneficial, primarily to address comorbidities which may be perpetuating factors (209). However, given somatic hypervigilance that can be found in some patients with FD and related FMDs, it may be advisable to first ensure that the patient is connecting to physical rehabilitation and psychotherapy recommendations before secondarily re-visiting psychotropic medication treatment. That being said, for patients with high acuity (and active) psychiatric comorbidities, it is advisable to involve a psychiatrist early in the assessment process for active management of psychiatric comorbidities. Particularly severe cases may also benefit from inpatient treatment, as part of a specific inpatient multidisciplinary treatment protocol (210).

Examination under sedation is also uniquely useful in cases of fixed functional dystonia, as relaxation under anesthesia (whether general or local anesthesia) suggests reversibility of symptoms, and can even be therapeutic (184): in one study, 5 out of 11 patients who did not respond to other treatments showed major improvements after anesthesia (183). A subset of FD patients may also respond to botulinum toxin injections, either as a placebo response, or by reducing tension in tonically activated muscles associated with fixed postures; however, unlike in OD, response may be immediate, which is not compatible with the pharmacological effect, which begins after a few days, typically peaking at 2 weeks (183, 211). While use of targeted anti-depressant or anxiolytic treatment, or treatment of neuropathic pain may be beneficial in FD, other typical medications used in the treatment of dystonia (benzodiazepines, trihexyphenidyl and other anti-cholinergics, or carbidopa/levodopa) and certainly DBS, should be avoided in FD (212, 213). Such treatments can have placebo effects but their potential risks far outweigh any benefits.

## Prognosis in Functional Dystonia

The prognosis for patients with FD is unfortunately generally poor (11). It is not uncommon for patients to experience no improvement in distress or functional impairment even after multiple courses of treatment (58, 79). Patients may also experience fluctuation in the severity of their symptoms, with only short-lived remissions (211). In some cases, treatment is altogether unsuccessful and symptoms can be progressive and debilitating (211, 214) and as many as 31% of patients may have worsening symptoms following treatment (211). Worse outcomes are particularly associated with the presence of chronic pain and CRPS (211). In a study assessing prognosis in fixed dystonia, there were higher levels of comorbid depression and somatic symptoms in those who had unchanged or worse symptoms on follow-up (211). Patient satisfaction with their physician also contributes to their outcomes (79); a retrospective study on FMD prognosis (40% FD), 22.1% noted worse outcomes, with patient dissatisfaction noted as a significant factor (214). There is also inconsistency and disagreement regarding how to assess patient outcomes in FD and other FMD, making it challenging objectively quantify treatment response and may result in suboptimal treatment (215).

Despite poor or variable responses to treatment, certain patients do improve, and in some cases undergo complete remission (79, 214). In a retrospective study assessing prognosis in FMD, 56.6% reported symptomatic improvement, while 43.4% had either no change or progression (214). Such positive outcomes in FMD have been associated with better physical and social health, as well as an overall shorter duration of illness (79, 214). In comparison, in a study of 35 fixed FD patients, 23% improved but only 6% had major remissions (211). Given a lack of a gold standard of management in patients, despite consensus guidelines, treatment must be individualized, tailored to the type of physical symptoms, and level of psychiatric comorbidity, with physical rehabilitation, psychotherapy and management of comorbidities as the primary components of the therapeutic multidisciplinary approach (183).

## Conclusions

FD is a diagnostically challenging and frequently debilitating subtype of FMD that can present in many different forms. Recognition of a FND in a patient presenting with dystonia symptoms is of paramount importance and we include a comprehensive appraisal of clinical clues and historical indicators of an FD diagnosis to help the practicing clinician. Adjunctive neurophysiological and other testing do not reliably differentiate FD from OD. A multidisciplinary approach, tailored to the patient, including neurology, psychiatry, PT, OT, speech therapy, and psychotherapeutic approaches is frequently required, in addition to possible use of botulinum toxin injections, other pharmacological approaches, and inpatient rehabilitation programs. Our understanding regarding the nature of FD and particularly post-traumatic dystonia is still evolving. Early diagnosis and treatment may help prevent unnecessary investigations and procedures, while facilitating the appropriate management of these highly complex patients.

## Author Contributions

LF: first draft of manuscript and acquisition of data. DP and NS: analysis and interpretation, and critical revision of the manuscript for important intellectual content. JC, JM, and PS: acquisition of data, analysis and interpretation, and critical revision of the manuscript for important intellectual content. CS: study concept and design, first draft of manuscript, acquisition of data, analysis and interpretation, and critical revision of the manuscript for important intellectual content. All authors contributed to the article and approved the submitted version.

## Permissions

Signed consent for the publishing of photographs was acquired for the use of all anonymous patient images in Figure 2.

## Conflict of Interest

DP has received honoraria for continuing medical education lectures in functional neurological disorder.NS received financial support from John Wiley & Sons for serving as editor in chief to Brain & Behavior. CS has provided scientific advisory for Xenon Pharmaceuticals and SwanBio Pharma and received research funding from Sanofi-Genzyme for a study of video oculography in late-onset GM2 gangliosidosis. He has received financial support from Sanofi-Genzyme, Biogen and Biohaven for the conduct of clinical trials. The remaining author declares that the research was conducted in the absence of any commercial or financial relationships that could be construed as a potential conflict of interest.
