# Supplementary material for: Functional Dystonia: Differentiation From Primary Dystonia and Multidisciplinary Treatments
Source: Front Neurol. 2021 Feb 4;11:605262. doi: 10.3389/fneur.2020.605262 (PMC7894256; doi:10.3389/fneur.2020.605262)
Supplement: Supplementary file 1 [file Table_1.docx]

**Supplemental material**

**Supplemental Table 1. Clinical Clues for Functional Dystonia Subtypes.**

| **FD body region/semiology** | **Clinical clues** |
| --- | --- |
| **General** | - Movement not patterned/stereotyped and is inconsistent and incongruent with other forms of dystonia (such as a mismatch between perceived and examination abilities and vice versa)*  - Multiple different and varying semiologies*  - Foot dystonia in an adult* (100)  - Dystonia at rest at onset* (100)  - Lack of overflow dystonia*  - Reduced or absent co-contraction of antagonistic muscles compared to other forms of dystonia (but with overlap with OD) (61)  - Distractibility* - Unusual/atypical sensory tricks*, such as sudden benefit with the application of a tuning fork  - Immediate placebo response to botulinum toxin injections*  - Other functional neurological signs (give-way/collapsing weakness, functional/midline-splitting sensory loss)*  - “Whack-a-mole” sign (102)*, where moving out of one abnormal posture can result in spread to the contralateral side or flit into another abnormal posture*  - Caution: there may commonly be hypertrophy and palpable spasm of affected muscles owing to excessive use, given frequent or fixed posturing*# |
| **Limb (general)** | - Fixed dystonia early in course (not to be confused with CBS, where this typically starts on the left or non-dominant side, sometimes with superimposed myoclonus)*  - Frequently painful on attempted movement of the affected limb  - Fixed dystonia may be associated with chronic regional pain syndrome type 1* (99)  - Active resistance against passive movement*#  - Tremor or clonus-like movement when evaluating range of motion or reflex testing of the limb but without any superimposed spasticity or hyperreflexia  - Straightening out posture or passively moving into a different posture can cause this to become a new resting fixed posture (akin to waxy flexibility of catatonia)  - Sensory symptoms, particularly anesthesia of the affected limb  - Ponderous and deliberate, slowed movements on affected side and can appear very effortful |
| **Lower limb** | - Typical semiology of plantarflexion and inversion with toe curling*  - Functional striatal toe sign* (101)  - Persistence of abnormal posture when walking backwards  - If bilateral, can involve a “frog-legged” posture  - Dragging leg behind and static abnormal posture throughout gait cycle  - Paradoxical improvement with stress gait or walking in tandem  - Appearance of weakness on movement away from the dystonic direction  - Excessive signs of effort in contrast to observed severity of gait disorder – the “huffing and puffing” sign  - Sensory symptoms particularly anesthesia of the foot  - Straightening out posture or passively moving into a different posture can cause this to become a new resting fixed posture (akin to waxy flexibility of catatonia) |
| **Upper limb** | - Typical semiology is fisting posture and wrist flexion, occasionally also elbow flexion or flexion of fingers at MCP or at IP joints*  - Ring/Little fingers commonly affected; Thumb least affected*  - Posture resolves with contralateral Luria sequencing  - Normal spontaneous movements in an affected limb (putting on glasses, using phone) |
| **Cervical** | - Varying semiology (i.e. changing from torticollis to laterocollis etc.) and may change when doing certain tasks  - Fixed neck posture, without a clear spasmodic nature  - Unusual, erratic and sometimes high amplitude associated tremor  - Active range of movement not possible during examination to despite spontaneous normal head movement at other times  - Lack of typical sensory tricks, such as resting head against a surface or lying flat  - Resolution of posture when writing or performing other tasks requiring concentration |
| **Trunk/gait** | - Inappropriate difficulties standing from sitting in comparison to gait  - Fixed posturing when walking, either extension or flexion at the waist  - Posture resolves when adjusting clothing or when doing a repetitive task (clicking fingers, tapping)  - Associated knee bending or unusual dromedary gait in an adult patient  - Posture does not improve when resting against a wall  - Associated episodic knee buckling  - Unusual relieving maneuvers (e.g. standing on one leg)  - Early use of crutches or other walking aid despite objective stability  - Associated prominent back pain  - Associated functional gait features (astasia abasia, unusual Romberg, unusual retropulsion pull testing), which may be at odds with apparent preserved balance when walking |
| **Blepharospasm/Meige** | - Gentle eye closure without spasms (pseudoptosis)*  - Forceful eye closure with episodes of normal eye opening*  - When eyes fully open, blink rate is normal  - When asked to actively and forcefully close eyes, does not provoke spasms (in sharp contrast to other forms of blepharospasm) with sudden, normal eye opening when opening  - Asymmetry and alternating spasms  - Eye/facial movements entrain with finger tapping or other hand movements  - Functional sensory symptoms/signs of the face, such as midline splitting or differential vibration sensation across frontal bone  - Can resolve with speech tasks requiring concentration (months of the year backwards, serials 7s etc.)  - Tonic spasms both involving eye closure and facial grimacing*  - Lower facial spasms resolve when talking or when testing tongue movements |
| **Hemifacial spasm** | - Lack of other Babinski sign* (124)  - Painful spasms or facial pain in general*  - Very forceful spasms, lasting longer than typical hemifacial spasm*  - Alternating spasms from one side to the other  - Lower face involvement at onset*  - Lower face more involved  - Tonic spasm with periods of normal facial movement in between  - Lack of synchronicity between upper/lower face  - Episodes of bilateral facial contraction* (104)  - Sensory symptoms of the face, particularly on the affected side, such as midline splitting or differential vibration sensation  - Stuck facial expression, which is variable during assessment  - Associated with stuttering/functional speech  - Normalizes or enhanced when testing eye movements or other facial movements |
| **Paroxysmal** | - Onset in adulthood (above age 20)*  - Associated tremor*  - Non-stereotyped episodes which are highly variable in semiology*  - Attacks precipitated by examination*  - Altered awareness during episodes (may overlap with functional / dissociative seizures)*  - Atypical triggers* (loud noises, light touch, startle etc)  - Absence of typical triggers (alcohol, caffeine, prolonged exercise etc)  - Unusual relieving maneuvers* (certain posture changes etc, touching a particular part of the body, massaging limb, passive range of motion), with sudden resolution  - Paroxysmal fixed posturing  - Additional FMD associated with episodes*  - Atypical response to medication with rapid, complete resolution*  - Stuttering functional speech during an episode |
| **Oromandibular** | - Ipsilateral downward and lateral jaw pulling (103)  - Complete resolution with certain facial movements, for instance whistling (caution for paradoxical improvement in certain oral mandibular OD) (105) |
| **Tongue** | - Tonic posturing  - If associated give-way weakness, deviation towards the side of facial weakness (“wrong way” sign) (103)  - Normal speech despite clearly involving the tongue  - Resolution of tongue involvement with testing individual speech sounds (lingual, labial and guttural) and holding prolonged phonation (“Ahh”, “Ee”) or even deep breathing  - Full range and normal active tongue movements despite deviation at rest  - Tongue moves in direction of eyes when testing eye movements |
| **Functional Spasmodic Dysphonia** | - Typically has a whispering, breathy quality* (100) or a persistent strained quality that is absent of voice breaks and spasms  - Breathy, high-pitched falsetto, or diplophonia (two tones)* (107)  - Lack of vocal tremor and rare vocal arrest* (100)  - Presence of a globus sensation*  - Speech accompanied by effortful and exaggerated facial, lip, tongue, neck, or respiratory movements  - Episodes of normal phonation with automatic activities (laughing, coughing, yawning), nonpropositional activities (counting, singing) and with facilitated distraction* (107)  - Speech may normalize when trying to count as far as the patient can in a single breath as they near the end of the breath  - Laryngoscopy reveals uniform hyperfunction across voice tasks, no phonetic variability and worsening at the end of a breath; false vocal fold constriction* (106) |

* Previously published (16, 39, 52, 99-107)

# Caution - can also be seen in primary dystonia at rest, particularly when the contractions are intense and forceful.
